# Specific Metabolomics Adaptations Define a Differential Regional Vulnerability in the Adult Human Cerebral Cortex

**DOI:** 10.3389/fnmol.2016.00138

**Published:** 2016-12-08

**Authors:** Rosanna Cabré, Mariona Jové, Alba Naudí, Victoria Ayala, Gerard Piñol-Ripoll, Maria P. Gil-Villar, Mayelin Dominguez-Gonzalez, Èlia Obis, Rebeca Berdun, Natalia Mota-Martorell, Manuel Portero-Otin, Isidre Ferrer, Reinald Pamplona

**Affiliations:** ^1^Department of Experimental Medicine, University of Lleida-Institute for Research in Biomedicine of Lleida (UdL-IRBLleida)Lleida, Spain; ^2^Neurology Division, Hospital Santa María of LleidaLleida, Spain; ^3^Institute of Neuropathology, University of BarcelonaBarcelona, Spain; ^4^Center for Biomedical Research on Neurodegenerative Diseases, Instituto de Salud Carlos III - ISCIIIBarcelona, Spain

**Keywords:** energy metabolism, mammalian target of rapamycin (mTOR), metabolomics, methionine cycle, mitochondrial stress, nucleotide metabolism, one-carbon metabolism, selective neuronal vulnerability

## Abstract

Brain neurons offer diverse responses to stresses and detrimental factors during development and aging, and as a result of both neurodegenerative and neuropsychiatric disorders. This multiplicity of responses can be ascribed to the great diversity among neuronal populations. Here we have determined the metabolomic profile of three healthy adult human brain regions—entorhinal cortex, hippocampus, and frontal cortex—using mass spectrometry-based technologies. Our results show the existence of a lessened energy demand, mitochondrial stress, and lower one-carbon metabolism (particularly restricted to the methionine cycle) specifically in frontal cortex. These findings, along with the better antioxidant capacity and lower mTOR signaling also seen in frontal cortex, suggest that this brain region is especially resistant to stress compared to the entorhinal cortex and hippocampus, which are more vulnerable regions. Globally, our results show the presence of specific metabolomics adaptations in three mature, healthy human brain regions, confirming the existence of cross-regional differences in cell vulnerability in the human cerebral cortex.

## Introduction

Human evolution is associated with rapid expansion of brain size and complexity, a prerequisite for the emergence of cognitive functions. These evolutionary changes have been linked to and supported by adaptations in brain metabolism, especially with respect to increased energy supply (Mink et al., 1981; Cáceres et al., 2003; Uddin et al., 2004; Fu et al., 2011; Somel et al., 2013). Thus, neurons in the human nervous system can perform a wide array of motor, sensory, regulatory, behavioral, and cognitive functions. This functional diversity is expressed in the central nervous system (CNS) by a complex organization in different regions that groups neuronal populations with a diversity of neural cells. The morphological and functional diversity among neurons suggests that each neuron type, and by extension each brain region, has its own genomic expression profile in addition to the ‘housekeeping’ genes necessary for the basal function of all cells, which are essentially related to cellular metabolism (Lein et al., 2007; Hawrylycz et al., 2012). This gene expression profile determines a proteomic pattern which, in turn, configures a regional neuron-specific metabolomic profile. Because each level of organization of the ‘-omics’ depends on the other, and a perturbation in one network can affect another, the phenotypic properties of different brain regions are ultimately the product of distinctive combinations of expressed gene products and their regulation, resulting in the metabolome as the informative modality to define cellular diversity in the CNS.

The fact that specific regions of the CNS exhibit differential vulnerabilities to aging and various neurodegenerative (NDD) and neuropsychiatric diseases (NPD) also reinforces the idea of the heterogeneity in neuronal responses to cell-damaging processes, in addition to specificity in the etiology of each pathology (Mattson and Magnus, 2006; Domínguez et al., 2016). So, in order to better understand the mechanisms which are involved in neuronal resistance/sensitivity to stress and death, it is crucial to define the cell vulnerability of the different brain regions in physiological conditions.

This study focuses on the prospects that an ‘omic’ approach offers for the identification of traits that define the selective neuronal vulnerability (SNV) for a given brain region, and their potential involvement in the neuronal aging process and the development of NDD and NPD. To date no metabolomic studies investigating cross-regional differences in the human brain have been reported. To overcome this limitation, here we use mass spectrometry-based technology (ESI-TQ-MS/MS) to measure the concentrations of 37 specific metabolites of three different regions of the adult human cerebral cortex.

We designed a panel of metabolites mostly belonging to the one-carbon metabolism, as an integrative network of nutrient status and energy metabolism which involves three pathways: the folate cycle, the methionine cycle, and the *trans*-sulfuration pathway (Locasale, 2013). In addition to the metabolomic analysis, we have also measured, using western blot, different factors associated with stress resistance and cell survival such as the antioxidants catalase and SOD1, the FOXO transcriptional factor FOXO1, the repressor element 1-silencing transcription factor REST (Lu et al., 2014), and the master regulator that senses cell nutrient and energy status, the mechanistic target of rapamycin mTOR (Perluigi et al., 2015).

Using this approach, we studied metabolic differences in three functionally and evolutionarily distinct brain regions: entorhinal cortex, hippocampus, and frontal cortex. The frontal cortex is a brain region that appeared recently during primate evolution and which is implicated in complex associative functions, while the entorhinal-hippocampus system functions as a hub in a widespread network for memory. We also focused on hippocampus, entorhinal and frontal cortex areas because of their importance in aging, NDDs such as Alzheimer’s disease (AD), and NPDs such as schizophrenia. This allowed us to examine how resistance to stress determines region-specific vulnerability in the adult human cerebral cortex.

## Subjects/Materials and Methods

### Chemicals

Unless otherwise specified, all reagents were from Sigma–Aldrich, and of the highest purity available.

### Human Samples

Brain samples were obtained from the Institute of Neuropathology Brain Bank following the guidelines of the local ethics committee, and in accordance with recently published criteria of sample quality (Ferrer, 2015). The study of human samples was carried out according to the Spanish Law of Science and accompanying guidelines and with the approval of the local ethics committee of the Bellvitge University Hospital (Barcelona, Spain). The selection of cases examined in the present study corresponded to a consecutive series of donations (see **Table 1**). The brains of adult healthy subjects were obtained at from 3 to 20 h after death, and were immediately prepared for morphological and biochemical studies, as previously described Ferrer et al. (2008).

**Table 1:** Cases examined.

Case	Age (*y*)	Gender	Post-mortem delay	Cause of death	Entorhinal cortex (*n* = 11)	Hippocampus (*n* = 9)	Frontal cortex (*n* = 11)
1	43	Male	4 h 35 min	Respiratory failure			x
2	43	Male	5 h 55 min	Multiorgan failure			x
3	47	Male	4 h 55 min	Cardiac arrest			x
4	48	Female	4 h	Respiratory failure			x
5	50	Male	17 h 15 min	Cardiac arrest	x		
6	52	Male	4 h 04 min	Myocardial infarction	x		
7	52	Male	4 h 40 min	Broncho-pneumonia			x
8	53	Male	7 h 25 min	Heart failure			x
9	54	Female	14 h 25 min	Bilateral pneumonia	x	x	
10	54	Male	10 h 35 min	Pneumonia	x	x	
11	56	Male	3 h 45 min	Renal failure	x	x	x
12	56	Male	8 h 50 min	Myocardial infarction	x	x	
13	57	Male	20 h 30 min	Respiratory failure	x	x	
14	58	Male	3 h 10 min	Massive intestinal ischaemia	x	x	
15	58	Male	4 h	Respiratory failure			x
16	58	Male	8 h 05 min	Pneumonia	x	x	
17	59	Male	4 h 15 min	Multiorgan failure	x	x	
18	61	Male	3 h 55 min	Multiorgan failure		x	x
19	64	Female	5 h	Heart failure			x
20	66	Female	4 h 15 min	Respiratory failure	x		x
Mean post-mortem delay (in hours)					9.01 ± 1.81	8.61 ± 1.94	4.76 ± 0.32#
Mean age^∗^ (in years)					56.36 ± 1.26	57.00 ± 0.76	53.72 ± 2.41


*^∗^Values are expressed as mean ± SEM. Non-significant differences were observed for the variable ‘age’ between regions. Non-significant differences were observed for ‘post-mortem delay’ between regions, with the exception of the comparison entorhinal cortex vs frontal cortex (#*p* = 0.048).*

Briefly, at autopsy, one hemisphere was fixed in 4% buffered formalin for about 3 weeks while the other hemisphere was cut in coronal sections 1 cm thick. Selected samples of the brain were dissected and placed in labeled plastic bags, immediately frozen on dry ice, and stored at -80°C until use. The neuropathological study was carried out on formalin-fixed, paraffin-embedded samples of the frontal, primary motor, primary sensory, parietal, temporal superior, temporal inferior, anterior cingulated, anterior insular, and primary and associative visual cortices; entorhinal cortex and hippocampus; caudate, putamen, and globus pallidus; medial and posterior thalamus; subthalamus; Meynert nucleus; amygdala; midbrain (two levels), pons, and medulla oblongata; and cerebellar cortex and dentate nucleus. De-waxed sections, 5-μm thick, were stained with haematoxylin and eosin, and Klü Barrera or processed for immunohistochemistry to β-amyloid, phosphorylated tau, α-synuclein, ubiquitin, p62, TDP43, glial fibrillary protein, and microglia markers.

Selected cases did not show lesions on the neuropathological examination including any kind of β-amyloid, tau, hypoxic, or vascular pathology. Following the initial screening, the present series includes 20 cases: 16 men and 4 women, aged from 43 to 66 years with post-mortem delay ranging from 3 h 10 min to 20 h 30 min (**Table 1**). Frozen samples of the entorhinal cortex (*n* = 11), hippocampus (*n* = 9), and frontal cortex area 8 (*n* = 11) were used for metabolomics and western blot studies. Samples from the three regions were processed in parallel.

### Metabolomic Analysis

An important technical concern is the accuracy of metabolite measurements made in postmortem brain tissue for *in vivo* metabolite concentrations. The criteria applied for the selection of cases ensure the quality of the samples and the preservation of the concentration of *in vivo* metabolites measured. Reinforcing this, previous studies demonstrated that the concentration of several metabolites (such as myo-inositol, creatine, glutamine, glutamate, *N*-acetylaspartate, taurine, spermine, spermidine, and putrescine) remained stable in postmortem brain tissue over long-term intervals (Perry et al., 1981; Petroff et al., 1988; Michaelis et al., 1996; Chen et al., 2009; Opstad et al., 2010). Because most of these metabolites belong to metabolic pathways associated with one-carbon metabolites, we understand that metabolites analyzed in the present study are stable; consequently, we excluded postmortem delay as a confounding factor in the present study.

#### Metabolite Extraction from Brain Samples

Tissue samples (40 mg) were homogenized in cold methanol (20 v/w) containing 1 μg/mL of phenylalanine C13 as internal standard and 1 μM butylhydroxytoluene as antioxidant, obtaining a final concentration of 50 mg tissue/mL. Then, samples were incubated at 20°C for 1 h and centrifuged at 12000 *g* for 3 min, and the supernatants were subjected to mass spectrometry analysis.

#### Triple Quadrupole Mass Spectrometry

For analysis, we have developed a new method (using a targeted approach based on LC ESI-TQ MS/MS) to detect and quantify a metabolomic panel including 37 metabolites belonging to energy metabolism and one-carbon metabolism in human brain tissue (see **Table 2**). Samples were decoded and randomized before injection. Every 5 samples, internal and external standards were injected as a quality control. Data were finally normalized according to deuterated internal standard content, and expressed as MS counts.

**Table 2:** Analytical traits of the panel of metabolites designed to be measured in the samples of cerebral cortex from healthy adults.

Dynamic MRM								

**Compound name**	**Precursor ion**	**Product ion**	**Ret time (min)**	**Delta ret time**	**Fragmentor**	**Collision energy**	**Cell accelerator voltage**	**Polarity**
3 P-Glycerate (^∗^)	184.9	96.9	0.82	1	75	12	7	Negative
*Cis*-Aconitate (^∗^)	175.03	139	1.5	1	100	15	7	Positive
Fumarate	115	71	1.8	1	60	4	7	Negative
Glutamic acid	146	102.1	0.8	1	75	12	7	Negative
Glutamine	147.1	84	0.79	1	70	16	7	Positive
Glyceraldehyde 3P	168.99	96.9	0.8	1	119	4	7	Negative
Leucine	132.1	90.3	0.9	1	82	12	7	Positive
L-Carnitine	162.1	60.1	0.81	1	107	16	7	Positive
NADH (^∗^)	666.9	136	2.4	1	124	44	7	Positive
NADPH (^∗^)	746.1	302	1.57	1	129	36	7	Positive
Phosphoenolpyruvate (^∗^)	166.97	78.9	0.84	1	55	8	7	Negative
Proline	116.07	70.1	0.81	1	75	16	7	Positive
Pyruvate (^∗^)	87	43.1	1.4	1	35	4	7	Negative
Succinate	117	73	2.2	1	65	8	7	Negative
Tryptophan	205.1	188	9.8	1	70	4	7	Positive
Betaine	118.09	58.1	0.82	1	107	28	7	Positive
Choline	105.12	61.1	0.78	1	92	16	7	Positive
Glycine	76	48.1	0.78	1	35	0	7	Positive
L-Serine	106.05	60.1	0.79	1	60	8	7	Positive
Sarcosine	90.06	44.1	0.8	1	40	16	7	Positive
Threonine	120.1	74.1	0.8	1	65	4	7	Positive
5-Methyl-THF (^∗^)	460.2	313.1	13.3	2	104	12	7	Positive
Cysteine	122.03	59	0.85	1	129	24	7	Positive
Cystathionine	223.08	88	0.8	1	77	24	7	Positive
Folate acid (^∗^)	442.15	295.1	9.35	2	92	8	7	Positive
GSH	308.09	84	1.6	1	97	28	7	Positive
GSSG (^∗^)	613.16	355	1.4	1	161	16	7	Positive
Homocysteine	136	90	0.88	1	65	4	7	Positive
Methionine	150.06	56.1	1.4	1	70	12	7	Positive
PLP (^∗^)	248	94	1.6	1	110	28	7	Positive
Pyridoxal	168.1	150	1.6	1	70	8	7	Positive
Pyridoxamine	169.1	134	0.82	1	100	20	7	Positive
SAH (^∗^)	385.13	136	4.39	1	97	12	7	Positive
SAM	399.15	250	0.8	1	100	8	7	Positive
Spermidine	146.2	72.1	0.71	1	75	12	7	Positive
Taurine	124	80	0.8	1	102	20	7	Negative
Adenosine	268.1	136	6	1	92	12	7	Positive
ADP (^∗^)	426.02	158.9	1.33	1	134	20	7	Negative
AMP	348.07	136	1.8	1	102	16	7	Positive
Deoxyguanosine	268.1	43.1	9.3	1	168	56	7	Positive
Deoxyguanosine 5MP (^∗^)	348.07	152	3.58	1	85	8	7	Positive
Guanine (^∗^)	152	135	1.46	1	107	16	7	Positive
Guanosine 5MP	364	152	0.86	1	87	8	7	Positive
Hypoxanthine	137.05	55.1	2	1	109	32	7	Positive
Inosine	269.09	137	7.3	1	70	4	7	Positive
Inosine 5MP	349.06	137	2.08	1	72	4	7	Positive
Inosine 5DP (^∗^)	427	158.9	1.2	2	119	24	7	Negative
Ribose 5-P	231.02	79.1	0.67	1	100	15	7	Negative
Xanthine	153	110	2.54	1	92	16	7	Positive
Xanthosine	285.1	153	7.6	2	65	4	7	Positive
Myo-inositol	179.05	87	0.75	1	102	16	7	Negative
*N*-Acetyl-Asp-Glut	305.1	148	2.9	1	70	4	7	Positive
*N*-Acetyl-Asp acid (^∗^)	174	88	1.49	1	75	12	7	Positive
Phenilalanine-^13^C (standard)	167.09	120.1	5.9	2	70	8	7	Positive


*Of the initial 53 metabolites included in and optimized for the analysis, 16 (marked by ^∗^) were finally excluded because they were absent after the extraction method was applied. So the final analyzed panel was composed of 37 metabolites.*

Samples were analyzed with liquid chromatography (UPLC 1290, Agilent Technologies, San Jose, CA, USA) coupled with electrospray ionization on a triple quadrupole mass spectrometer (ESI-TQ MS/MS, Agilent Technologies 6420, San Jose, CA, USA). For analysis 6 μL of the extract was injected. Chromatographic separation was achieved on a reversed phase C18 (2.1 × 50 mm, 1.8 μm particles; Agilent Technologies, San Jose, CA, USA) column using a flow rate of 0.2 mL/min during a 19 min gradient (0–5 min 0% B, 5–8 min from 0% B to 30% B, 8–9 min from 30% B to 100% B, 8–12 min 100% B, 12–13 min from 100% B to 0% B, 13–19 min 0% B), while using the solvents A, 0.1% formic acid, and B, acetonitril 0.1% formic acid. Electrospray ionization was performed in both positive and negative ion mode (depending on the target metabolite) using N_2_ at a pressure of 50 psi for the nebulizer with a flow of 12 L/min and a temperature of 325°C, respectively.

To detect the individual metabolites, multiple reaction monitoring (MRM) in negative and in positive ion mode was performed with individually optimized fragmentor voltage and collision energies (Optimizer Application, MassHunter, Agilent Technologies, San Jose, CA, USA). Most of the MRM parameters were achieved by flow injection of pure standards and the MassHunter Optimizer software (Agilent Technologies, San Jose, CA, USA). However, some of metabolites required manual optimization using MassHunter Qualitative Analyses (Agilent Technologies, San Jose, CA, USA). All the MRM parameters obtained from optimization were compared to the literature when available for certain compounds. Finally, a chromatographic system was applied to determine retention time of each standard. Peak determination and peak area integration were carried out with MassHunter Qualitative Analyses (Agilent Technologies, San Jose, CA, USA).

### Mass Spectrometry Analysis of 2-SC

2-SC was determined as trifluoroacetic acid methyl ester (TFAME) derivatives in acid-hydrolysed, delipidated, and reduced brain protein samples with GC/MS using a HP6890 Series II gas chromatograph (Agilent, Barcelona, Spain) with an MSD5973A Series detector and a 7683 Series automatic injector, an HP-5MS column (30 m × 0.25 mm × 0.25 μm), and the described temperature program (Naudí et al., 2013). Quantification was performed with internal and external standardization using standard curves constructed from mixtures of deuterated and non-deuterated standards. Analyses were carried out with selected ion-monitoring GC/MS (SIM-GC/MS). The ions used were lysine and [^2^H_8_]lysine, m/z 180 and 187, respectively, and 2-SC and [^2^H_2_]SC, m/z 284 and 286. The amount of product was expressed as μmoles of 2-SC per mol of lysine.

### Western Blot Analysis

The amounts of different factors associated with stress resistance and cell survival such as the antioxidants catalase and SOD1, the FOXO transcriptional factor FOXO1, the repressor element 1-silencing transcription factor REST, and the master regulator that senses cell nutrient and energy status, mechanistic target of rapamycin mTOR, were estimated using western blot analyses in samples from brain tissue.

Brain tissue (50 mg from each specific brain region) was homogenized in a buffer containing 180 mM KCl, 5 mM MOPS, 2 mM EDTA, 1 mM diethylenetriaminepentaacetic acid, 1 μM butylated hydroxyltoluene, protease inhibitor mix (80-6501-23, Amersham Biosciences), and phosphatase inhibitors (Na3VO4 1 mM, NaF 1 mM). A brief centrifugation (1000 rpm at 4°C for 3 min) to pellet and remove cellular debris was performed. The protein concentration was measured using the Bradford method (Bio-Rad Protein Assay 500-0006). Proteins were separated with one-dimensional SDS-PAGE. Samples were mixed with sample buffer (62.5 mM Tris–HCl pH 6.8, 2% SDS, 10% glycerol, 20% 2-β-mercaptoethanol and 0.02% bromophenol blue) and heated at 95°C for 5 min. Proteins (35 μg) were subjected to electrophoresis on 10% SDS-polyacrylamide minigels. For immunodetection, proteins were transferred, using a Mini Trans-Blot Transfer Cell (Bio Rad) in a buffer containing 25 mM TRIS, 192 mM Glycine, and 20% methanol, to polyvinylidene difluoride (PVDF) membranes (Immobilon-P Millipore, Bedford, MA, USA). The membranes were immersed in blocking solution (0.5% BSA Sigma-Aldrich A4503, 0.1% Tween in TBS) at room temperature for 1 h. After blocking, the membranes were washed two times using 0.05% TBS-T buffer. Afterward, they were incubated in primary solution using specific antibodies: anti-GFAP (1:1000, ref. ab7260), anti-catalase (1:1000, ref. ab16731), anti-SOD1 (1:5000, ref. ab52950), anti-FoxO1 (1:1000, ref. 2880 Cell Signaling), anti-REST (1:1000, ref. ab21635), and anti-phospho-mTOR and anti-mTOR (1:1000 in both cases, ref. 2971s and 2972-Cell Signaling Technology, respectively). An antibody to actin (1:5000, ref. A5441 Sigma) was also used in each analysis to determine the amount of the different factors in reference to total protein mass. Primary antibody specificity was tested by incubating only with the secondary antibody.

The primary antibody was incubated at 4°C for 16 h. Then, the membrane was washed three times in 0.05% TBS-T buffer and incubated at room temperature for 1 h with the appropriate secondary antibodies [ECL Anti-mouse IgG, horseradish Peroxidase linked whole antibody-NA93IV GE Healthcare (1:50000) and ImmunoPure Goat Anti-Rabbit IgG peroxidase conjugated-31460 Pierce Biotechnology (1:100000)]. After five washes with 0.05% TBS-T buffer, bands were visualized using an enhanced chemiluminescence HRP substrate (Millipore, Bedford, MA, USA). Signal quantification and recording was performed with ChemiDoc equipment (Bio-Rad Laboratories, Inc., Barcelona, Spain). The amounts of the determined factors were specifically calculated from the ratio of their densitometry values in reference to the densitometry values of their own actin content. Ratio of phospho-mTOR to total-mTOR was calculated. The amounts of REST and GFAP were specifically calculated from the ratio of their densitometry values in reference to the densitometry values of their gallyas stain protein.

### Statistical Analysis

All statistic calculations were performed using the SPSS software (SPSS Inc, Chicago, IL, USA). Values were expressed as means ± standard error of the mean (SEM). Comparisons between groups were made with ANOVA followed by DMS tests for paired groups. The minimum level of statistical significance was set at *p* < 0.05 in all the analyses.

## Results

**FIGURE 1:**
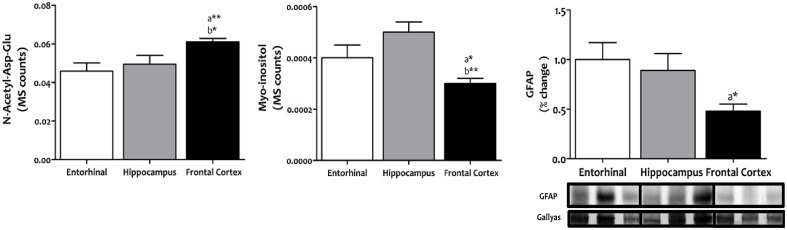
**Content of neurons and glial cells in different regions of the adult human cerebral cortex.** *N*-acetyl-Asp-Glu was used as a marker for neuronal content, and myoinositol and GFAP as markers for glial cell content. *N*-acetyl-Asp-Glu and myoinositol were determined with TQMS, while GFAP was measured with western blot. ^∗^*p* < 0.05; ^∗∗^*p* < 0.01; ^∗∗∗^*p* < 0.001. a, significant differences with respect to entorhinal cortex; b, significant differences with respect to hippocampus.

Evidence from comparative studies of gene expression and evolution between humans and anthropoid primate species suggests that neurons from human neocortex are characterized by high energy metabolism, along with an increase in neuroglial cell density in order to support greater metabolic demands (Sherwood et al., 2006). We have extended this idea to three different regions of the adult human brain cerebral cortex to evaluate possible differences in the density of glial cells relative to neurons, as an indirect indication of region-specific adaptation to neuronal metabolic demands. Our results demonstrate that frontal cortex shows an increased density of neurons along with a concomitant decrease in glial cells compared to entorhinal cortex (*p* < 0.01 and *p* < 0.05, respectively) and hippocampus (*p* < 0.05 and *p* < 0.01, respectively) (**Figure 1**), suggesting lower neuronal metabolic demands specifically in frontal cortex.

**FIGURE 2:**
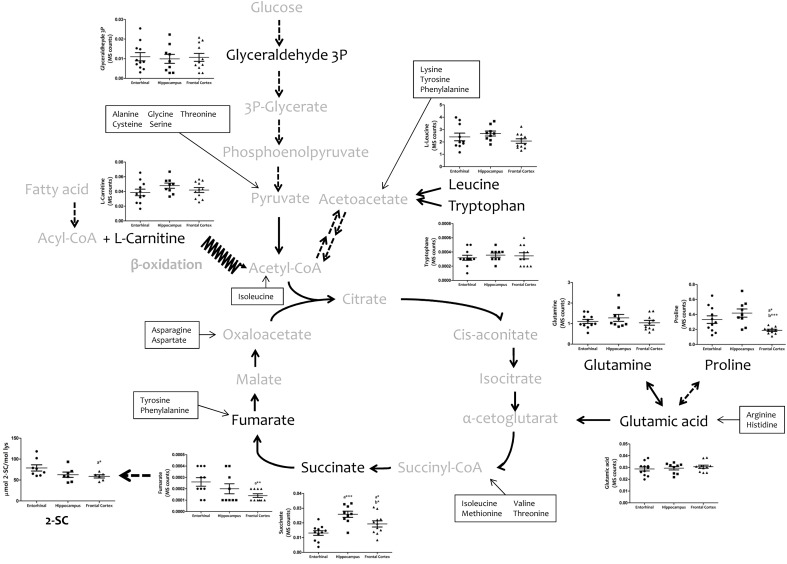
**Concentrations of metabolites involved in bioenergetics metabolism in different regions of the adult human cerebral cortex.** Metabolites were detected and quantified with TQMS. The steady-state level of 2-SC was measured with GC-MS. ^∗^*p* < 0.05; ^∗∗^*p* < 0.01; ^∗∗∗^*p* < 0.001. a, significant differences with respect to entorhinal cortex; b, significant differences with respect to hippocampus.

To explore the bioenergetic demands of the different regions of the human cerebral cortex, we measured, using a TQMS approach, a myriad of metabolites which directly (succinate and fumarate) or indirectly (leucine, tryptophan, glutamate, glutamine, proline, carnitine, and glyceraldehyde-3-phosphate) are associated with or involved in Krebs cycle (**Figure 2**). No interregional differences were detected for leucine, tryptophan, carnitine, glutamate, or glutamine. In contrast, the concentrations of succinate and fumarate, and of proline, were significantly lower in frontal cortex compared to entorhinal cortex and hippocampus, while no differences were observed between entorhinal cortex and hippocampus.

Since mitochondria play a key role in cell bioenergetics, we detected and measured 2-SC as a biomarker of mitochondrial stress in order to test for potential cross-regional differences. 2-SC [S-(2-succino)cysteine] is a chemical modification of cysteine in proteins by the Krebs cycle intermediate, fumarate, via a succination reaction. Recent studies suggest that succination is a mechanistic link between mitochondrial dysfunction, oxidative and ER stress, and cellular progression toward apoptosis (Merkley et al., 2014). Our results show that the steady-state levels of 2-SC are significantly lower in frontal cortex compared to entorhinal cortex, with no differences between entorhinal cortex and hippocampus (**Figure 2**).

**FIGURE 3:**
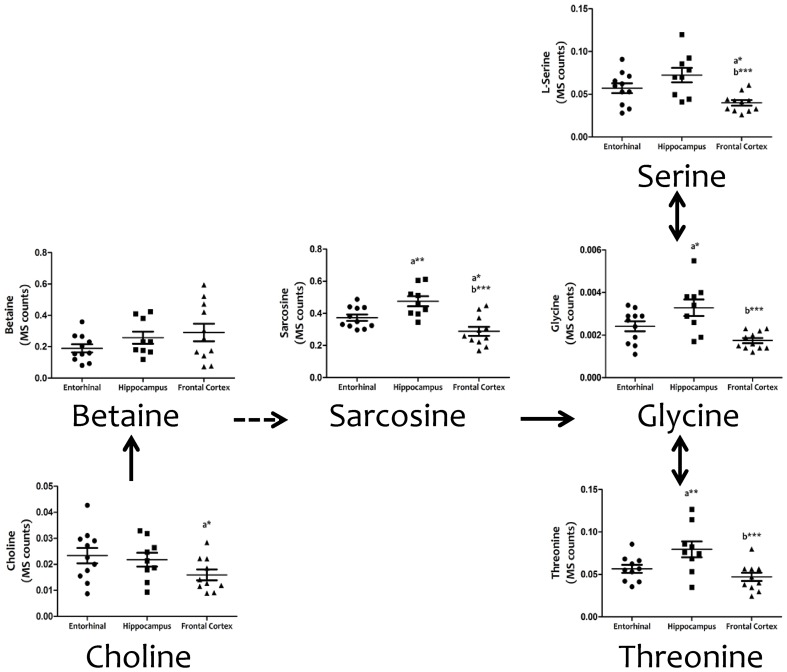
**Tissue concentrations of metabolites from the serine, glycine and threonine metabolism in different regions of the adult human cerebral cortex.** All metabolites were measured with TQMS. ^∗^*p* < 0.05; ^∗∗^*p* < 0.01; ^∗∗∗^*p* < 0.001. a, significant differences with respect to entorhinal cortex; b, significant differences with respect to hippocampus.

Cell physiology requires the biosynthesis of a diversity of cellular components (including proteins, lipids, and nucleic acids), as well the maintenance of cell redox status, and genetic and epigenetic status. Amino acid metabolism involving serine and glycine, and the carbon units that they provide, covers many of these requirements. **Figure 3** demonstrates the existence of significant inter-regional differences in the concentrations of serine, glycine, and threonine, and related metabolites such as choline, betaine, and sarcosine, thereby verifying that the concentrations of all these metabolites are significantly lower in frontal cortex, and higher in hippocampus, compared to entorhinal cortex.

**FIGURE 4:**
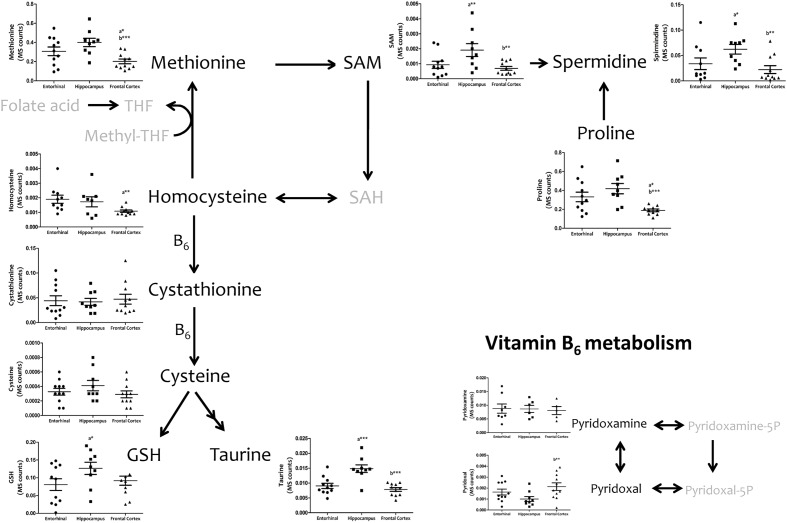
**Tissue concentrations of metabolites belonging to the methionine metabolism, including the methionine cycle and the *trans*-sulfuration pathway, in different regions of the adult human cerebral cortex.** All metabolites were measured with TQMS. ^∗^*p* < 0.05; ^∗∗^*p* < 0.01; ^∗∗∗^*p* < 0.001. a, significant differences with respect to entorhinal cortex; b, significant differences with respect to hippocampus.

**FIGURE 5:**
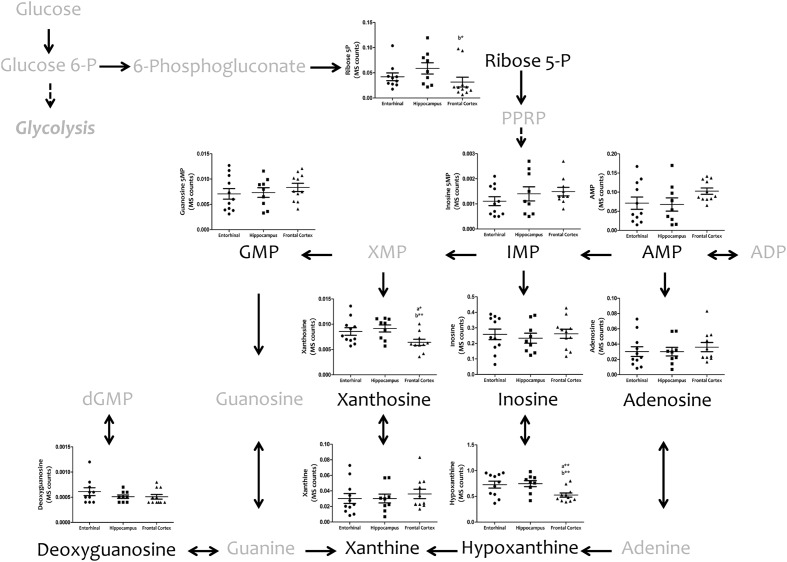
**Tissue concentrations of metabolites from the purine metabolism in different regions of the adult human cerebral cortex.** All metabolites were measured with TQMS. ^∗^*p* < 0.05; ^∗∗^*p* < 0.01; ^∗∗∗^*p* < 0.001. a, significant differences with respect to entorhinal cortex; b, significant differences with respect to hippocampus.

We then investigated interregional differences of one-carbon metabolism in human cerebral cortex. In particular, we focused our analysis on metabolites belonging to the methionine metabolism (including the methionine cycle and the *trans*-sulfuration pathway) and purine metabolism. To assess these pathways, we measured, with TQMS, the metabolites (directly or closely related to) that follow: (a) for methionine cycle: methionine, *S*-adenosyl-methionine, and homocysteine, as well as spermidine and proline; (b) for the *trans*-sulfuration pathway: cystationine, cysteine, gluthatione, taurine, and vitamin B6 (pyridoxal and pyridoxamine); and finally, (c) for purine metabolism: inosine monophosphate (IMP), adenosine monophosphate (AMP), guanosine monophosphate (GMP), adenosine, inosine, xanthosine, deoxyguanosine, hypoxanthine, and xanthine, and we included the metabolite from pentose phosphate pathway ribose-5-phosphate. TQMS analysis showed a marked decrease in the concentration of all metabolites of the methionine cycle in frontal cortex with respect to hippocampus and/or entorhinal cortex, while significantly higher concentrations of SAM and spermidine in hippocampus compared to entorhinal cortex were detected (**Figure 4**). No interregional changes were detected for metabolites of the *trans*-sulfuration pathway, with the exception of taurine, which was significantly higher in hippocampus compared to entorhinal cortex, and again lower in frontal cortex compared to hippocampus (**Figure 4**). No interregional differences were detected for the metabolites of the purine metabolism, with the exception of xanthosine and hypoxhantine, which were significantly lower in frontal cortex (**Figure 5**).

**FIGURE 6:**
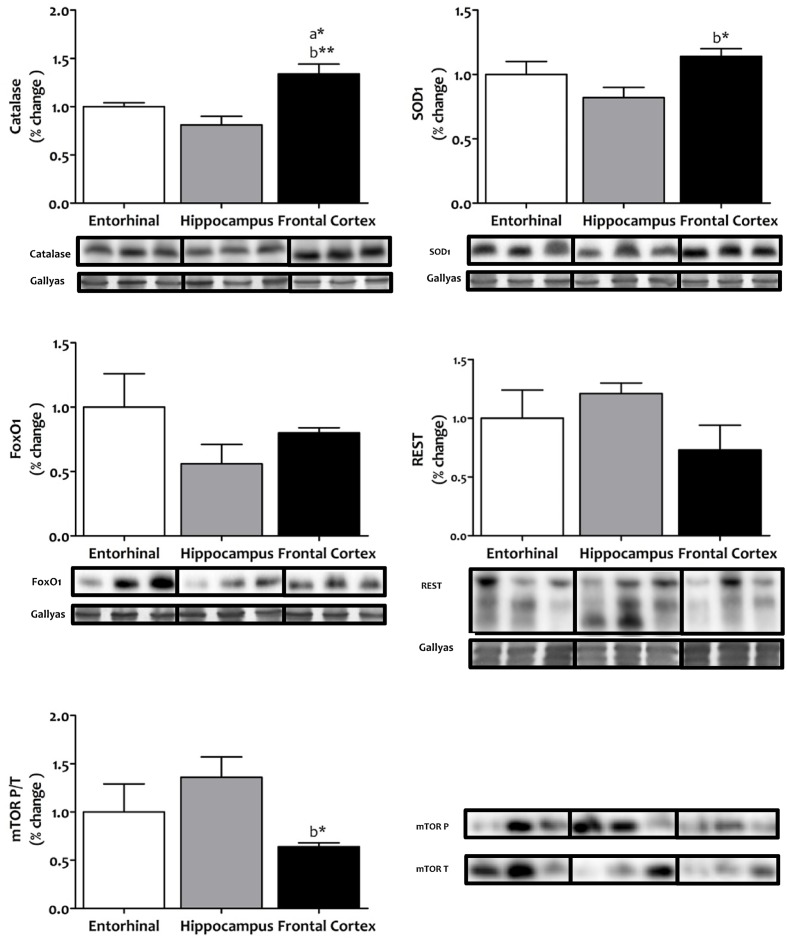
**Tissue protein expression of factors associated with stress resistance and cell survival in different regions of the adult human cerebral cortex.** The antioxidants catalase and SOD1, the FOXO transcriptional factor FOXO1, the repressor element 1-silencing transcription factor REST, and the master regulator that senses cell nutrient and energy status, mechanistic target of rapamycin mTOR, where all determined with western blot. ^∗^*p* < 0.05; ^∗∗^*p* < 0.01; ^∗∗∗^*p* < 0.001. a, significant differences with respect to entorhinal cortex; b, significant differences with respect to hippocampus. All western blots can be found as **Supplementary Figure S1**.

We then considered whether this interregionally differing metabolic status might be associated with changes in cellular systems linked to stress resistance and cell survival. To this end, we measured the antioxidant enzymes catalase and SOD1, the FOXO transcriptional factor FOXO1, the repressor element 1-silencing transcription factor REST, and the master regulator that senses cell nutrient and energy status, mechanistic target of rapamycin mTOR (**Figure 6**). Western blot analysis showed that catalase and SOD1 were significantly and specifically increased in frontal cortex, that FOXO1 and REST did not show interregional differences, and that the activation of mTOR was significantly lower in frontal cortex.

## Discussion

Although metabolic pathways important to brain function are conserved across diverse taxa (Peregrín-Alvarez et al., 2009), current findings show that brain metabolism experienced relevant changes in the human species (Somel et al., 2013). In addition to evolutionary considerations, brain neurons offer a diverse response to stresses during the physiological aging process or as a result of both NDD and NPD (Mattson and Magnus, 2006; Jové et al., 2014; Naudí et al., 2015). The morphological and functional diversity among neuronal cells, the temporal trajectory of functional losses during the aging process, and the temporal pattern and specificity in the appearance and development of each NDD and NPD, as well as the heterogeneity in neuronal responses to detrimental processes associated with each of the pathologies, all confirm the existence of a cross-regional SNV (Mattson and Magnus, 2006; Jové et al., 2014; Naudí et al., 2015). This SNV could be expressed through a neuron(region)-specific metabolomic profile. Hence, metabolomics can help to define and improve understanding of cellular (regional) diversity in the CNS. From an inter-regional comparative perspective there is, however, a lack of studies focused on outlining the specific metabolomics of the distinctly functional sub-regions of the brain. To shed light on this, we performed a comparative metabolomic analysis of three healthy human brain regions: entorhinal cortex, hippocampus, and frontal cortex.

Our results show the existence of reduced energy demand, mitochondrial stress, and one-carbon metabolism (particularly restricted to the methionine cycle) specifically in frontal cortex. These findings, along with a better antioxidant capacity and lower mTOR signaling as well in frontal cortex, suggest that this brain region is especially resistant to stress compared to the entorhinal cortex and hippocampus, which are more vulnerable regions.

The one-carbon metabolism can be considered as an integrative network of nutrient status. Thus, inputs in the form of amino acids (which donate carbon units) enter the metabolic network, are metabolized, and then become output for diverse biological functions which include biosynthesis of cell components, regulation of redox status, regulation of methylation reactions, and regulation of nucleotide pools. The partitioning of carbon units into these different cellular outputs basically involves three interconnected pathways: the folate cycle, the methionine cycle, and the *trans*-sulfuration pathway (Locasale, 2013). Several studies have shown that defects in one-carbon metabolism in brain induce deep disturbances in cell physiology as a consequence of the relevant pathways where one-carbon metabolism is involved, and also, more importantly, through the toxic effects derived from the metabolites which shape the core of the methionine cycle. Thus, a connection has been established between high levels of homocysteine and cognitive function, from mild cognitive decline to vascular dementia and AD (Miller, 2003). In contrast, low methionine and derived metabolite content, either constitutively or induced by nutritional intervention, is associated with resistance to stress and a longer lifespan (Pamplona and Barja, 2006, 2011; Naudí et al., 2007). Hence, we may infer that the lower one-carbon metabolism observed in frontal cortex is a physiological adaptation which confers resistance to stress on this region.

mTOR is a conserved serine/threonine kinase which regulates metabolism in response to nutrients, growth factors, and cellular energy conditions. Available evidence indicates that the mTOR signaling pathway is involved in brain aging and age-related NDD diseases (Garelick and Kennedy, 2011; Bockaert and Marin, 2015; Perluigi et al., 2015). In this line, an increasing number of studies show that disruption in mTOR signaling in the brain affects multiple pathways including glucose metabolism, energy production, mitochondrial function, and autophagy. Conversely, attenuation of the mTOR signal, through pharmacological or nutritional intervention, increases longevity and is associated with a healthy lifespan, including improvement in brain function (Garelick and Kennedy, 2011; Perluigi et al., 2015). Consequently, we may infer that the lower mTOR signaling observed in frontal cortex is a physiological adaptation which confers resistance to stress on this region.

## Conclusion

Our results define the existence of metabolomic differences in three different regions of the mature, healthy human brain, confirming the existence of cross-regional differences in the brain. We must note, however, that although our study covers key cellular metabolic pathways, it is far from being comprehensive. Nevertheless, our findings indicate that the metabolomic signature is an optimized feature associated with diversity among neuronal populations in brain cortex, allowing us to hypothesize that the metabolic optimization of some physiological traits, such as resistance to stress, is region-specific. However, it is evident that more studies are needed to draw a metabolomic-wide atlas of metabolites in the adult human brain.

## Author Contributions

IF and RP designed the experiments. RC, MJ, AN, MD-G, and RP analyzed the data. RC, VA, GP-R, MG-V, EO, RB, NM-M, and MP-O performed the experiments. RP supervised the design and data interpretation. The manuscript was written by IF and RP and edited by AN and RP. All authors discussed the results and commented on the manuscript.

## Conflict of Interest Statement

The authors declare that the research was conducted in the absence of any commercial or financial relationships that could be construed as a potential conflict of interest.

## Supplementary Material

**FIGURE S1 | Western blots of tissue protein expression of factors associated with stress resistance and cell survival in different regions of the adult human cerebral cortex.**
