# Supplementary figures and images for: Specific Metabolomics Adaptations Define a Differential Regional Vulnerability in the Adult Human Cerebral Cortex
Source: Front Mol Neurosci. 2016 Dec 8;9:138. doi: 10.3389/fnmol.2016.00138 (PMC5143679; doi:10.3389/fnmol.2016.00138)

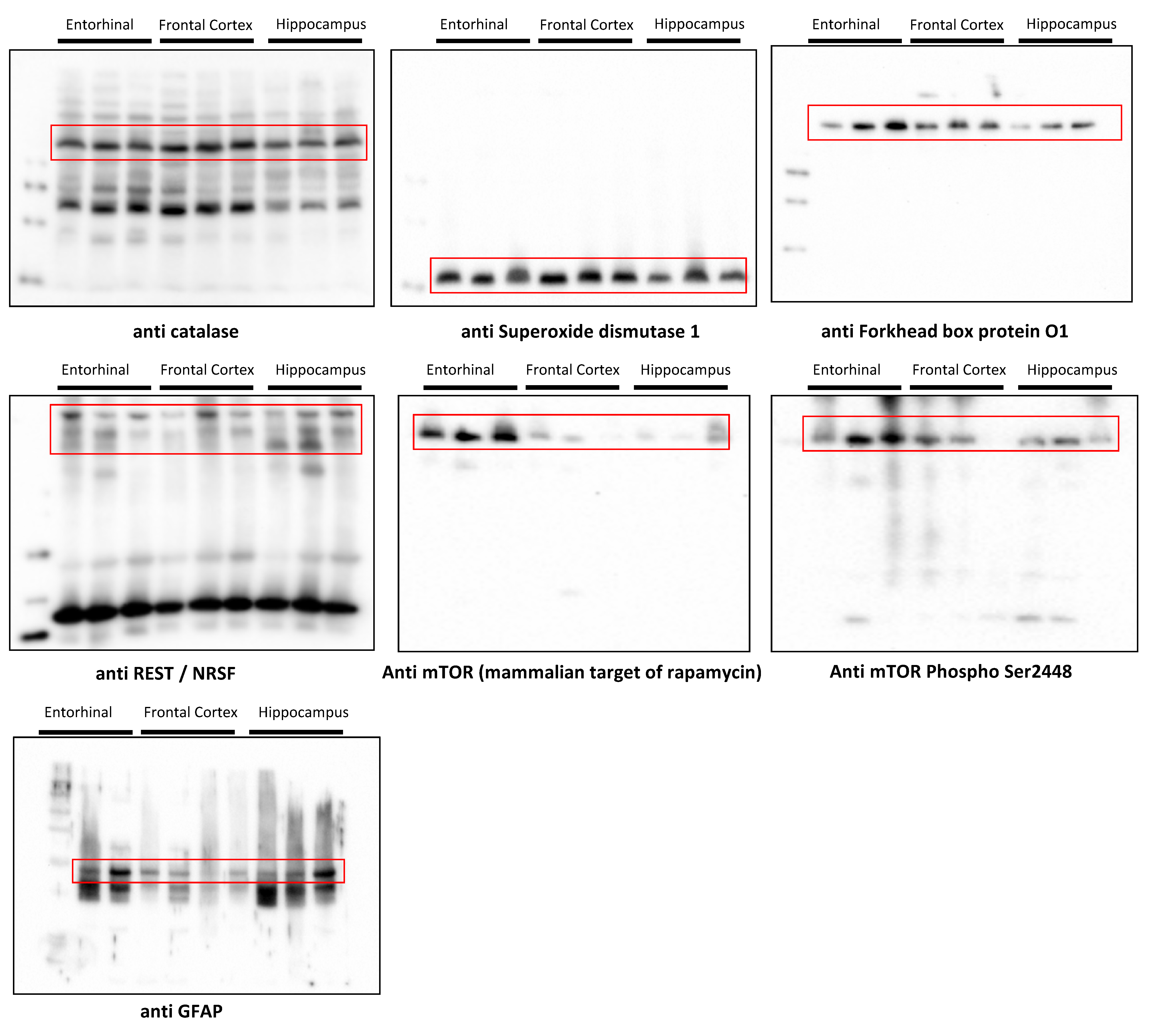

Supplement: Supplementary file 1 [file Image_1.TIF]
